# Global conservation prioritization areas in three dimensions of crocodilian diversity

**DOI:** 10.1038/s41598-023-28413-6

**Published:** 2023-02-13

**Authors:** Ricardo Lourenço-de-Moraes, Felipe S. Campos, Pedro Cabral, Thiago Silva-Soares, Yhuri C. Nobrega, Amanda C. Covre, Frederico G. R. França

**Affiliations:** 1 Programa de Pós-graduação em Ecologia e Monitoramento Ambiental (PPGEMA), Universidade Federal da Paraíba, Rio Tinto, PB 58297‐000 Brazil; 2 NOVA Information Management School (NOVA IMS), Universidade Nova de Lisboa, Campus de Campolide, 1070-312 Lisbon, Portugal; 3 Universitat Autònoma de Barcelona, 08193 Cerdanyola del Vallès, Catalunya Spain; 4 Centre de Recerca Ecològica i Aplicacions Forestals (CREAF), 08193 Cerdanyola del Vallès, Catalunya Spain; 5 Herpeto Capixaba project, Instituto Biodiversidade Neotropical, Nova Guarapari, Guarapari, ES 29206-400 Brazil; 6 Museu de História Natural do Sul do Estado do Espírito Santo, Universidade Federal do Espírito Santo, Jerônimo Monteiro, ES 29550-000 Brazil; 7 Projeto Caiman, Instituto Marcos Daniel, Vitória, ES 29055-290 Brazil; 8 Departamento de Medicina Veterinária, Centro Universitário FAESA, Vitória, ES 29053-360 Brazil; 9 Programa de Pós-graduacão em Ecologia de Ambientes Aquáticos Continentais (PEA), Universidade Estadual de Maringá, Maringá, PR 87020-900 Brazil

**Keywords:** Ecology, Conservation biology

## Abstract

Crocodilians are a taxonomic group of large predators with important ecological and evolutionary benefits for ecosystem functioning in the face of global change. Anthropogenic actions affect negatively crocodilians’ survival and more than half of the species are threatened with extinction worldwide. Here, we map and explore three dimensions of crocodilian diversity on a global scale. To highlight the ecological importance of crocodilians, we correlate the spatial distribution of species with the ecosystem services of nutrient retention in the world. We calculate the effectiveness of global protected networks in safeguarding crocodilian species and provide three prioritization models for conservation planning. Our results show the main hotspots of ecological and evolutionary values are in southern North, Central and South America, west-central Africa, northeastern India, and southeastern Asia. African species have the highest correlation to nutrient retention patterns. Twenty-five percent of the world’s crocodilian species are not significantly represented in the existing protected area networks. The most alarming cases are reported in northeastern India, eastern China, and west-central Africa, which include threatened species with low or non-significant representation in the protected area networks. Our highest conservation prioritization model targets southern North America, east-central Central America, northern South America, west-central Africa, northeastern India, eastern China, southern Laos, Cambodia, and some points in southeastern Asia. Our research provides a global prioritization scheme to protect multiple dimensions of crocodilian diversity for achieving effective conservation outcomes.

## Introduction

Carrying important evolutionary information, the Order Crocodylia appeared in the Late Cretaceous period and among the extant Archosauria, crocodilians remain as the closest sister group of birds^1^. Their long evolutionary history (i.e. over 200 my) has been driving the ecology and evolution of an enormous variety of species on Earth^2^. Crocodilians are the largest inhabitants of freshwater ecosystems and are highly exposed to anthropogenic pressures, mainly due to habitat loss^3^, with half of the living species threatened with extinction^4^. Despite limited empirical knowledge, they have been globally identified as potential bioindicators due to their sensitivity to pollution, and dependence on aquatic habitats^5–8^. Crocodilians are semiaquatic predators and may be of crucial importance in aquatic and terrestrial ecosystems^2,9^. These iconic animals have varied roles in the aquatic and terrestrial ecosystems as top-order predators, influence the nutrient cycle, and cross-ecosystem engineering processes^2,10,12^. Crocodilian species differ from other vertebrates by their demographic characteristics and can generally be categorized as K-selected, characterized by presenting a long life, often large size, and few offspring; or R-selected, characterized by presenting a short life span, many offspring, and usually small size^11^.

Crocodilians are considered one of the twenty groups most charismatic in the world^12^, attracting the public attention because of their morphological features, thus promoting marketing for financial resources for conservation plans^13^. Due to the need for good protection across a large number of different habitats, crocodilians can be considered flagship-umbrella species; act as flagship species because they are charismatic^13^ and act as an umbrella species because their conservation protects a large number of species that coexist in the same environment^14^.

Despite the increased conservation efforts, there is a gap in the literature that integrates ecological and evolutionary interactions that assess crocodilian species as mediators of cross-ecosystem linkages in the landscapes^2^. Effective conservation planning must protect taxonomic, functional and phylogenetic diversity, to ensure the persistence of all biodiversity components^15^. Taxonomic diversity (TD) is the number of species in a determinate area. Functional diversity (FD) is a dimension of diversity that represents the extent of ecological differences between species based on the distinction of their morphological, physiological, and life-history features^16^. Phylogenetic diversity (PD) adds value to theoretical and applied ecology studies, distinguishes species according to their evolutionary histories, and quantifies how much of the Tree of Life is represented locally^17^. Therefore, for any effective conservation plan, TD, FD and PD components should be considered as a central issue beyond the detailed knowledge of the species’ distribution^18^. Finding areas with multiple biodiversity components can be essential for the effectiveness and achievement of conservation goals^19^. Thus, prioritize areas covering a minimum area with more chances of success in maximizing biodiversity conservation.

Protected areas (PA) typically figure as the cornerstone of conservation strategies worldwide, covering about 15% of the Earth’s surface^20,21^. The current protected network is particularly helpful in safeguarding biodiversity, although far from enough in the face of habitat loss^22–24^. For improved conservation outcomes, mapping ecological and evolutionary values of different species pools may be key in determining the establishment of new PA in aquatic and terrestrial ecosystems^18,25^.

Given the ecological importance of crocodilians for cross-ecosystem fluxes, here we explore how their biodiversity components are distributed on Earth, and how they are correlated to nutrient retention patterns. For this, we calculate three dimensions of crocodilian diversity, and evaluate the effectiveness of the global PA networks in conserving species, thus suggesting three scenarios of conservation prioritization models. These three scenarios differ in the values of the different dimensions of diversity (TD, FD and PD) and the distribution range of threatened species. This work aims to contribute to conservation strategies focusing on the role of crocodilian species in ecosystem functioning worldwide.

## Results

**Figure 1:**
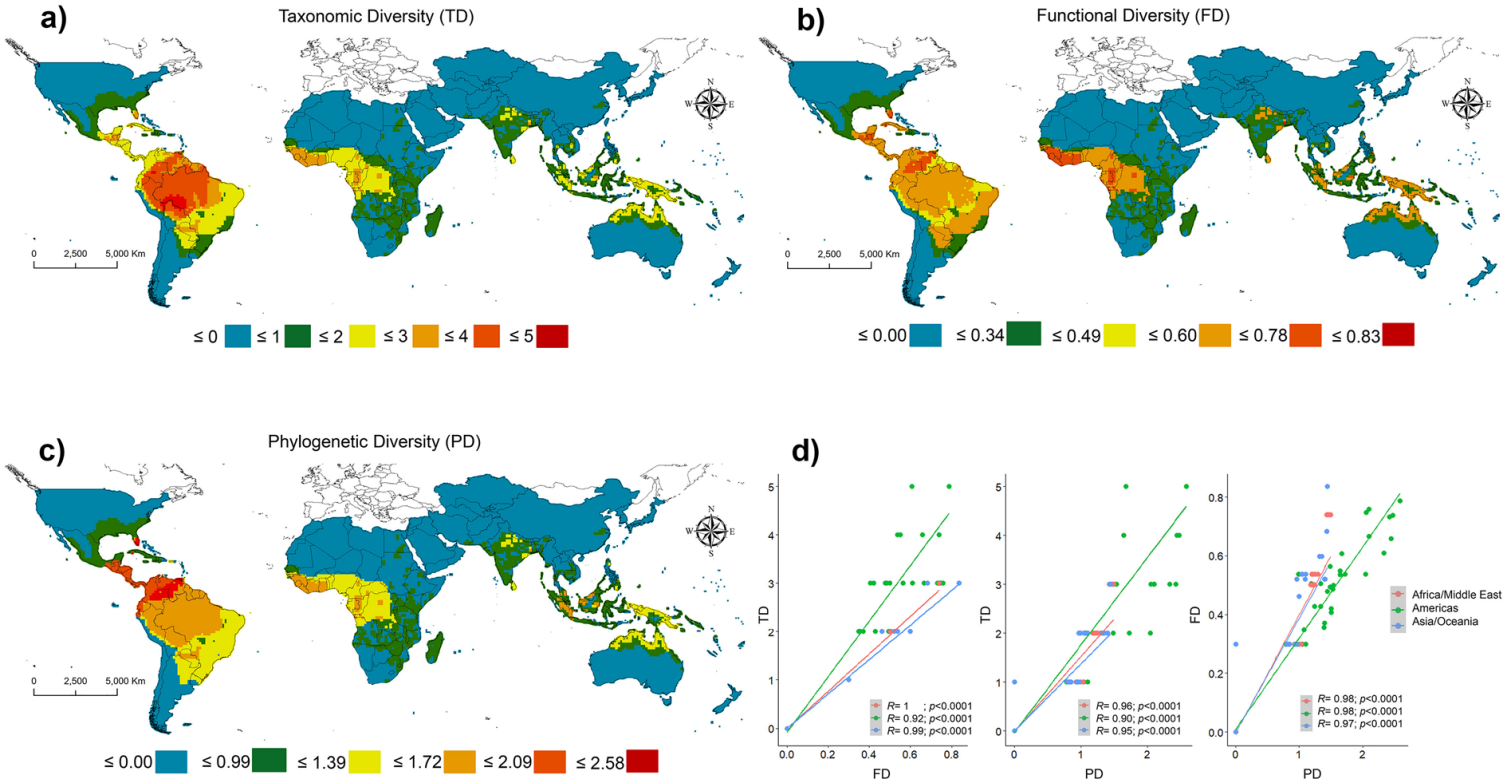
Spatial distribution of crocodilians worldwide. (**a**) Taxonomic diversity (TD); (**b**) functional diversity (FD); (**c**) phylogenetic diversity (PD); and (**d**) relationships between TD, FD and PD. The maps were created in ArcGIS Pro software^60^.

As they are ectothermic, crocodilians have distribution patterns related to elevated temperatures, which corresponds to a latitudinal range between − 30° and 20°. On the three dimensions of crocodilian diversity on Earth, our results show high values of TD (i.e. number of species per cell, see materials and methods), FD and PD in tropical and subtropical regions, at latitudes between − 15° and 20°. High TD and FD values are distributed in the north to central South America, central Central America, and southern North America. In Africa, high TD, FD and PD values are concentrated in the west-central region (Fig. 1a–c). In Asia, the main regions are located in northeastern India, Sri Lanka, Malaysia, western Indonesia, Borneo and New Guinea, northern and southern Philippines, and northern Oceania (Fig. 1a–c). The highest TD is in South America, mainly in the Amazon Basin (Fig. 1a) where occur five species in sympatry. The higher values of FD in the Americas are in extreme southern North America, central Central America, and extreme northern South America; in Africa, higher FD values are in the west-central and it is a small portion in the direction south of the west-central region (see Fig. 1c). The highest values of PD are distributed in the extreme southern North America and extreme northern South America, where in sympatry species of subfamily Alligatorinae (e.g. *Alligator, Caiman*) and Crocodylinae (e.g. *Crocodylus*) occur, with distinct evolutionary lineages (Fig. S2a).

The highest FD is in northeastern India, where occurs the *Gavialis gangeticus,* a species functionally distinct from the other crocodilian species (Fig. S2b). Higher values of TD, FD and PD are in Malaysia and Indonesia mainly in Borneo, Sumatra, north of Java, and New Guinea, and northern Oceania (Fig. 1). The null models for FD and PD show different values than expected by chance (*p* < 0.001), indicating a non-random pattern of FD and PD. The distribution of the three dimensions of crocodilian diversity through different landscape patterns on Earth shows a high spatial correlation between TD, FD and PD values (*R* = 0.90 to 1, *p* < 0.0001; Fig. 1d), highlighting their interaction of ecological and evolutionary scales, and the effects of these interactions on ecosystem-level processes.

**Figure 2:**
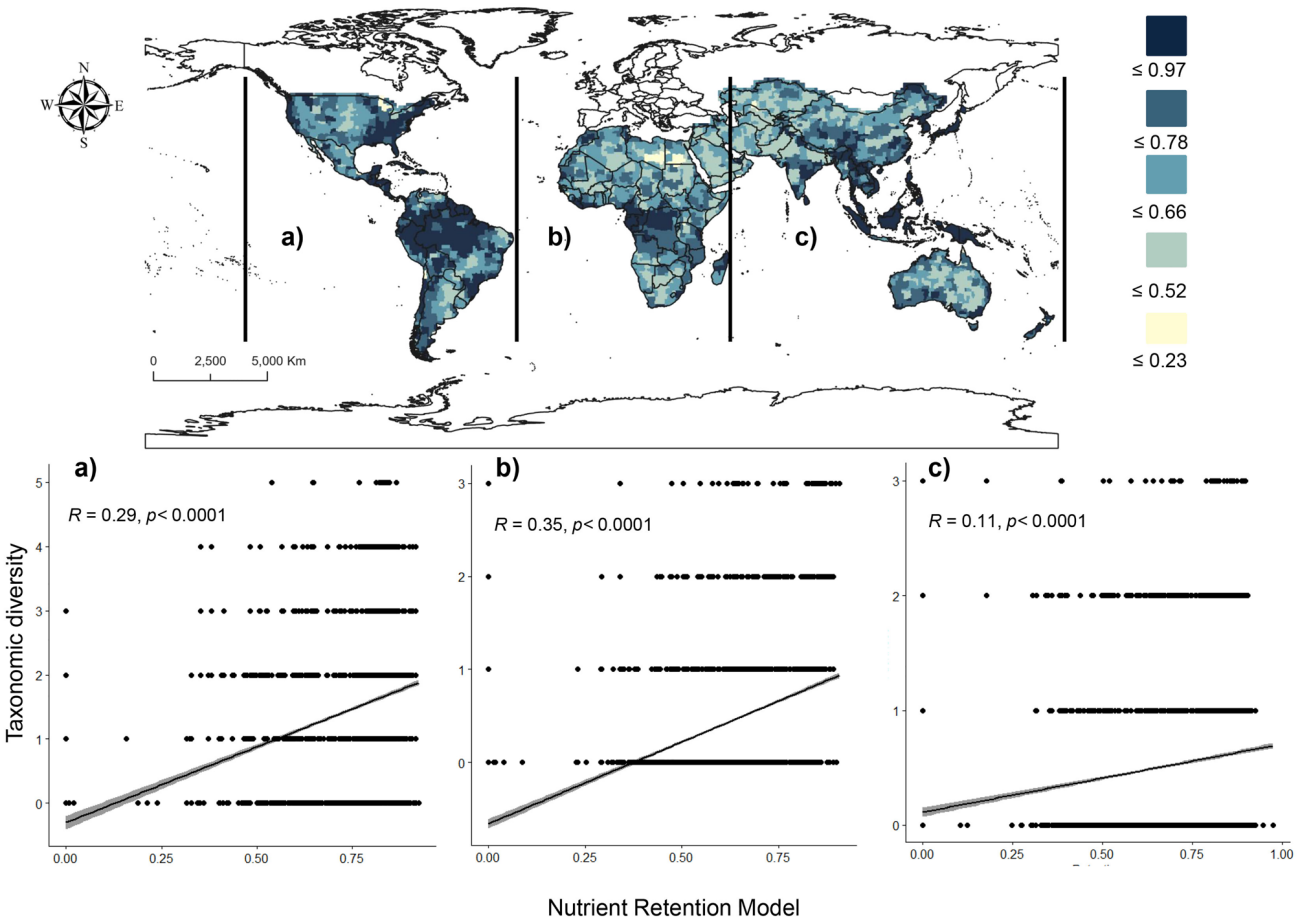
Spatial distribution on the global-scale of Nutrient Retention (Map). (**a**) Relationship between Nutrient Retention and Crocodilians’ TD in North, Central and South America; (**b**) relationship between Nutrient Retention and Crocodilians’ TD in Africa and the Middle East; (**c**) relationship between Nutrient Retention and Crocodilians’ TD in Asia and Oceania. The maps were created in ArcGIS Pro software^60^.

**Figure 3:**
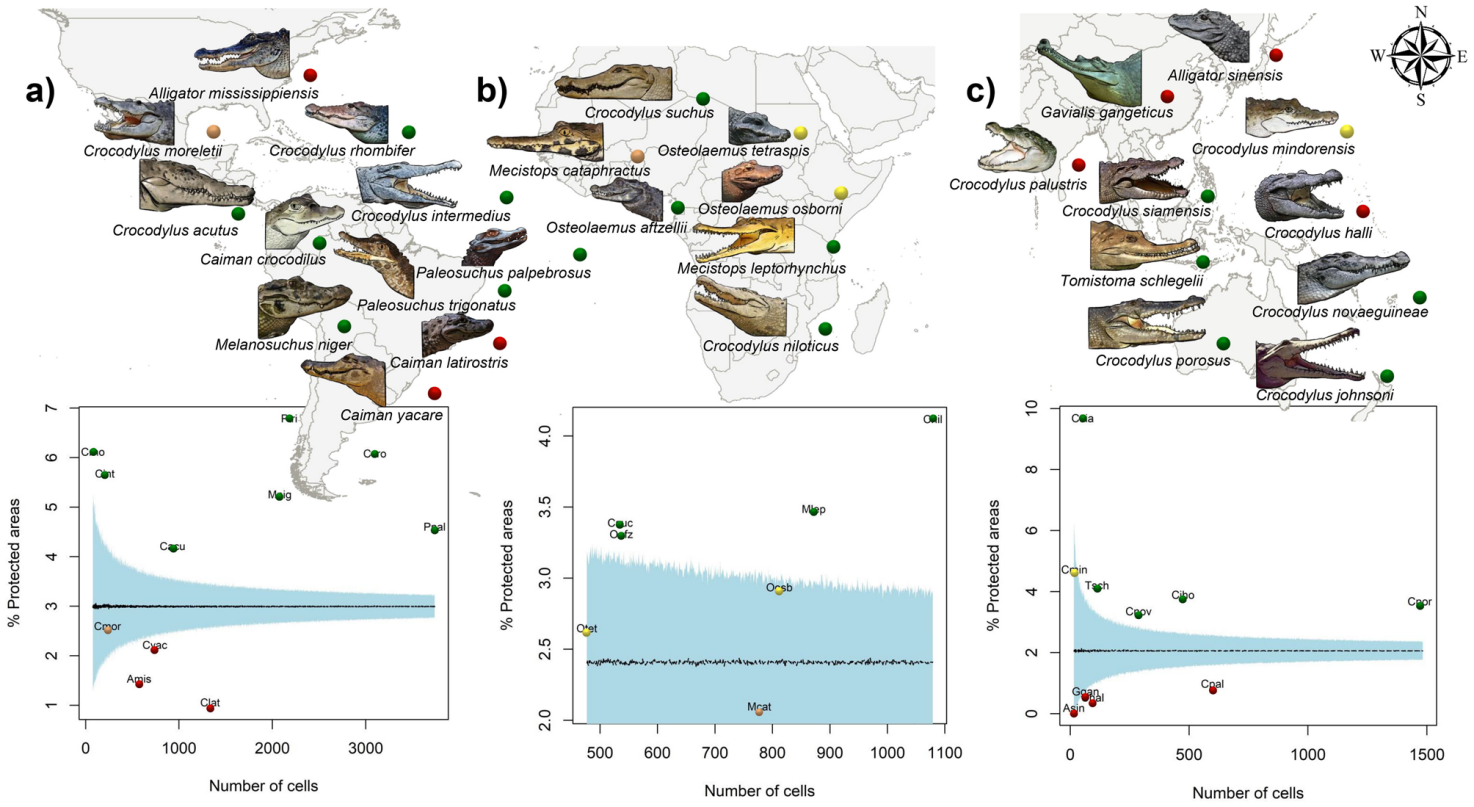
Representation of distribution of crocodilian species and spatial relationship of the Mean Percentage Overlap (MPO) between each species range and the global PA networks. Results of null models: green dots denote values significantly higher than expected by chance, red dots denote values significantly lower than expected by chance, and yellow (above mean) and orange (below mean) dots denote non-significant (*p* < 0.05) values. (**a**) Crocodilian species in North, Central and South America (Amis = *Alligator mississippiensis*, Cyac = *Caiman yacare*, Clat = *Ca. latirostris*, Ccro = *Ca. crocodilus*, Cacu = *Crocodylus acutus*, Cint = *Cr. intermedius*, Cmor = *Cr. moreletii*, Crho = *Cr. rhombifer*, Mnig = *Melanosuchus niger*, Ppal = *Paleosuchus palpebrosus*, Ptri = *P. trigonatus*). (**b**) Crocodilian species in Africa and the Middle East (Cnil = *Cr. niloticus*, Csuc = *Cr. suchus*, Mcat = *Mecistops cataphractus*, Mlep = *Me. leptorhynchus*, Otet = *Osteolaemus tetraspis,* Oaft = *O. aftezelli, Oosb* = *O. osborni*). (**c**) Crocodilian species in Asia and Oceania (Asin = *A. sinensis*, Chal = *Cr. halli*, Cjoh = *Cr. johnsoni*, Cmin = *Cr. mindorensis*, Cnov = *Cr. novaeguineae*, Cpal = *Cr. palustris*, Cpor = *Cr. porosus*, Csia = *Cr. siamensis*, Ggan = *Gavialis gangeticus*, Tsch = *Tomistoma schlegelii*). Dashed lines indicate the mean percentage overlap from 1,000 randomizations, and the light blue surface represents the random range, with 95% confidence interval. The maps were created in ArcGIS Pro software^60^ and the illustrations of species were created by L-d-M, Lia.

Our results show that Africa has the highest values of the positive interaction between TD and nutrient retention (35%), followed by the Americas (29%) and Asia and Oceania (11%) (Fig. 2). The mean percentage overlap (MPO) demonstrates that the PA networks cover an average of 57.1% of the species’ ranges currently protected (individual species ranging from 3.3 to 9.7%, SD ± 1.72%, Fig. 3, Table 1). In 17.8% of the species, the level of protection is not significantly different from that expected by chance. In 25% of the species (e.g. *A. mississippiensis, A.sinensis, Ca. latirostris*, *Ca. yacare, Cr. halli,* and *G. gangeticus*), the distribution patterns are significantly higher than expected by chance (Fig. 3, Table 1), with the lowest level of representativeness in the PA networks.

**Table 1:** Mean percentage of spatial overlap (MPO) between the range of crocodilian species and protected areas networks (IUCN Red List categories I to IV^4^) of the World.

Species	MPO observed	MPO randomized	Representativeness	IUCN
*Alligator mississippiensis*	1.432	2.983	−	LC
*A. sinensis*	0.000	2.037	−	CR
*Caiman crocodilus*	6.071	2.993	+	LC
*Ca. latirostris*	0.946	2.992	−	LC
*Ca. yacare*	2.107	2.993	−	LC
*Crocodylus acutus*	4.166	2.990	+	VU
*Cr. halli*	0.350	2.066	−	LC
*Cr. intermedius*	5.658	2.990	+	CR
*Cr. johnsoni*	3.745	2.078	+	LC
*Cr. mindorensis*	4.631	2.040	*	CR
*Cr. moreletii*	2.519	3.006	*	LC
*Cr. niloticus*	4.120	2.410	+	LC
*Cr. novaeguineae*	3.230	2.042	+	LC
*Cr. palustris*	0.685	2.061	−	VU
*Cr. porosus*	3.527	2.055	+	LC
*Cr. rhombifer*	6.109	2.968	+	CR
*Cr. siamensis*	9.692	2.050	+	CR
*Cr. suchus*	3.376	2.396	+	VU
*Gavialis gangeticus*	0.515	2.052	−	CR
*Mecistops cataphractus*	2.057	2.413	*	CR
*Me. leptorhynchus*	3.467	2.398	+	EN
*Melanosuchus niger*	5.206	2.995	+	NT
*Osteolaemus aftezelli*	3.294	2.393	+	EN
*O. osborni*	2.913	2.419	*	VU
*O. tetraspis*	2.618	2.403	*	VU
*Paleosuchus palpebrosus*	4.543	2.992	+	LC
*P. trigonatus*	6.793	2.983	+	LC
*Tomistoma schlegelii*	4.095	2.059	+	VU

Results of null models describing the representativeness of the species in protected areas: (−) denotes values significantly lower than expected by chance, (+) denotes values significantly higher than expected by chance, and (*) denotes non-significant (p < 0.05) values. IUCN Red List categories for all 28 crocodilian species: CR – Critically Endangered, EN – Endangered, LC – Least Concern, NT – Near Threatened, VU – Vulnerable.

**Figure 4:**
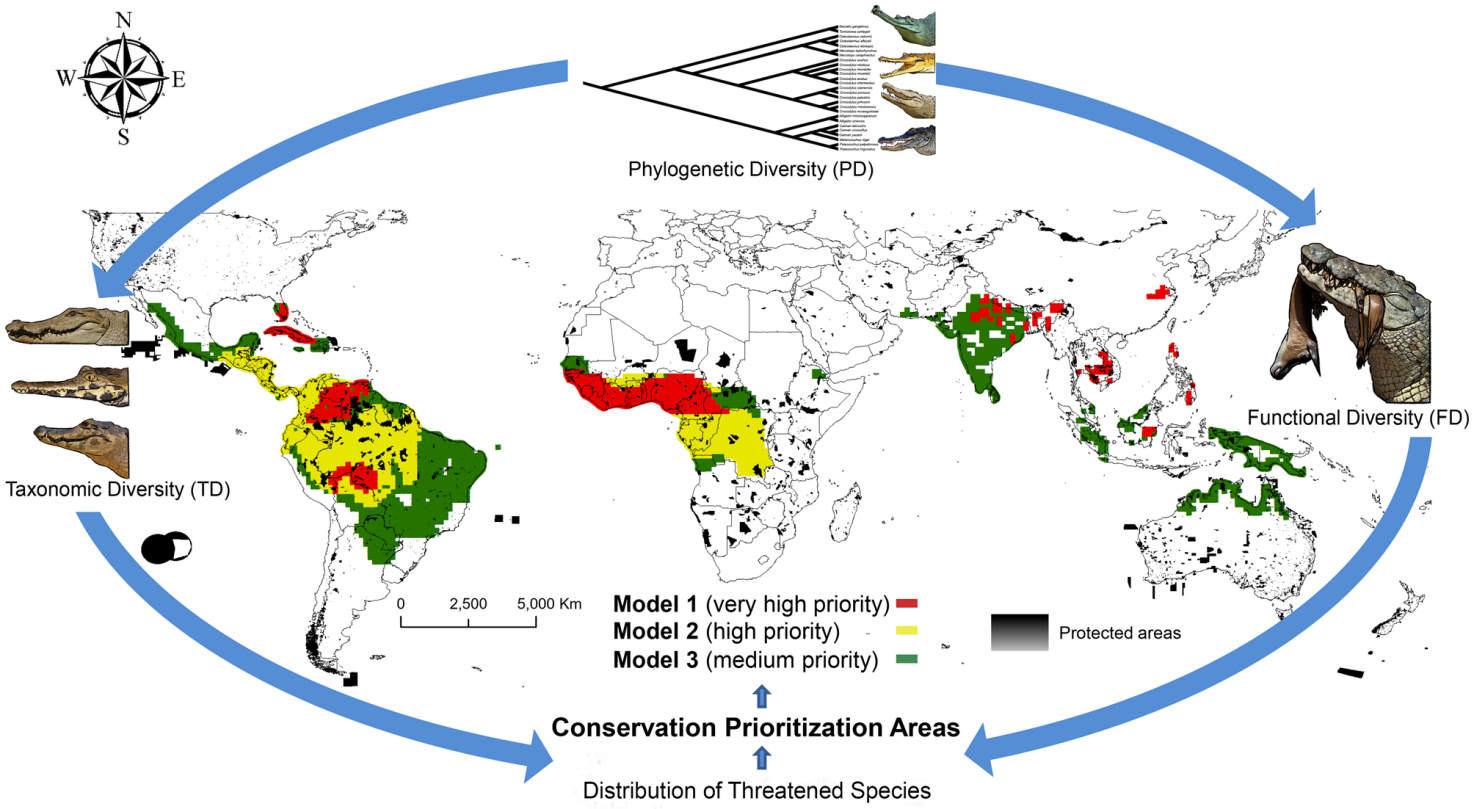
Spatial distribution of the conservation prioritization models on a global scale, based on the three dimensions of crocodilian diversity (TD, FD and PD) and spatial distribution of threatened species. The maps were created in ArcGIS Pro software^60^ and the illustrations of species were created by L-d-M. Lia.

The regions prioritized by Model 1(see Fig. 4, Table 2) hold values of FD, PD and TD higher than 90% of the total observed in the world and the presence of Critically Endangered (CR) species. The main conservation areas indicated by Model 1 (Fig. 4, Table 2) are southern North and east-central Central America, with one CR species (*Cr. rhombifer*) and high values of FD and PD; northern South America, with one CR species (*Cr. intermedius*), and in the Amazon basin with the highest values of TD and higher values of FD and PD; west-central Africa, with one CR species (*Mecistops cataphractus*), and high values of TD, FD and PD, having three species (*Me. cataphractus, Osteolaemus tetraspis, O. osborni*) not significantly protected in the PA networks (see Fig. 3). Northeastern India, southern Nepal, and Bangladesh also can be considered an important region for crocodilian conservation efforts, with one CR species (*G. gangeticus*), the highest values of FD, and high values of TD and PD. The eastern China also covers one CR species (*A. sinensis*), which is highly threatened with extinction, with their distribution patterns not represented in the PA networks (MPO = 0.00; Fig. 3, Table 1). In southeastern Asia, a little portion of southern Vietnam and south-central Thailand, southern Laos, Cambodia, northern and southern Philippines, and Borneo also cover two CR species (*Cr. mindorensis* and *Cr. siamensis*), with higher values of FD and high values of TD and PD.

**Table 2:** Areas with the highest priority for crocodilian conservation worldwide and their relative percentage by country.

Country	Area (km^2^)	Relative percentage (%)
Bangladesh	234,963.48	69.41
Benin	117,057.68	61.91
Bolivia	143,981.29	12.40
Brazil	618,214.55	6.98
Burkina Faso	12,068.24	4.01
Cambodia	258,627.70	82.64
Cameroon	509,056.90	84.55
Central African Republic	232,748.66	24.13
Chad	36,499.10	2.87
China	254,398.23	2.82
Colombia	640,940.08	56.50
Cuba	107,415.63	100.00
Democratic Republic of the Congo	36,859.72	1.61
Ghana	228,481.86	95.58
Guinea	317,132.00	92.96
Guinea-Bissau	19,072.10	100.00
Guyana	32,564.25	15.47
India	644,551.70	20.45
Indonesia	120,355.71	6.41
Ivory Coast	423,054.19	100.00
Laos	47,367.70	20.60
Liberia	120,610.98	100.00
Myanmar	35,114.95	5.26
Nepal	10,850.34	7.37
Niger	60,123.45	5.09
Nigeria	600,591.30	66.10
Peru	96,233.90	7.45
Philippines	105,439.76	36.06
Republic of the Gambia	110,856.15	65.31
Thailand	78,055.34	15.24
United States	62,452.63	0.85
Venezuela	493,966.75	54.45
Vietnam	49,391.12	15.23

Areas denote regions selected by Model 1 (i.e. FD, PD and TD higher than 90% of the total observed and with the presence of CR species^4^).

The regions prioritized by Model 2 (Fig. 4) hold values of FD, PD and TD higher than 70% of the total observed in the world and the presence of Endangered (EN) species. Model 2 prioritizes west-central Central America, north and central South America; west-central Africa with two EN species (*Me. leptorhynchus* and *O. aftezelli*). The regions prioritized by Model 3 (Fig. 4) hold values of FD, PD and TD higher than 50% of the total observed in the world and the presence of Vulnerable (VU) species. Model 3 shows important areas for crocodilian conservation in northern Central America and little portions of east-central Central America, southern North America, and north and south-central South America, with one VU species (*Cr. acutus*); west-central Africa and a little portions of northern Ethiopia, with three VU species (*Cr. suchus*, *O. osborni,* and *O. tetraspis*); a little portions of southeastern Iran, southern Pakistan, India, Sri Lanka, a little portions of southern Nepal, parts of Malaysia and Indonesia, and northern Oceania, with two VU species (*Cr. palustris* and *T. schlengelii*).

## Discussion

Our results show different patterns of TD, FD and PD around the world and show the importance of using the three dimensions of diversity for conservation strategies by aggregating the evolutionary history and ecology of species. South America showed the highest TD values, and southern North America and northern South America had the highest PD values. This pattern is probably due to the evolutionary history of crocodilians in the Americas with two lineages of phylogenetically distinct origins (i.e. Alligatoroidea and Gavialoidea). The species with Alligatoroidea form has a robust body, broad head and skulls, and low saltwater tolerance, the species that evolved in North America (genus *Alligator* is extant) dispersed from North to Central and South America diversifying^26^. Three genera are currently extant in South America (*Caiman*, *Melanosuchus,* and *Paleosuchus*^26^). Currently, two basal species of the subfamily Alligatorinae live in colder areas in southern North America (*A. mississippiensis*), and eastern China (*A. sinensis*). The other group is species with Gavialoidea forms that possibly arrived from the African continent and diversified from Central America to northern South America^27^. The Gavialoidea forms have a skull elongated and greater saltwater tolerance^27^, currently in America only the genus *Crocodylus* is extant. These two evolutionary lineages that diversified in the Americas showed the highest TD and PD values, and higher FD values.

Asia had the highest FD values, despite being concentrated in a small area in northeastern India. Although previously occurring over a wide geographic area, today the *G. gangeticus* occurs in small and fragmented areas in northeastern India, Nepal, and Bangladesh^4^. It is a species with distinctive functional traits (Fig. S2b), with a long thin skull, a large size as an adult, an aquatic life, and high functional value^28,29^. Another species that occurs in sympatry with the *G. gangeticus* in a small area in northeastern India is the *Cr. porosus*, a species with a high saltwater tolerance and one of the largest species at its adult size. These two species living in sympatry, together with *Cr. palustris*, provide the highest FD values in the world. In central Borneo where the species *T. schlegelii* with distinct functional characteristics, such as the long and thin skull, occurs in sympatry with *Cr. porosus* and *Cr. siamensis* promoting higher FD values. High values of PD occur in areas where the two of the most basal species of the subfamily Gavialinae live in northeastern India (*G. gangeticus*) and southeastern Asia (*T. schlegelii*) in sympatry with species of genus *Crocodylus* (subfamily Crocodylinae). In Africa, Gavialoidea forms have diversified, and currently, three distinct extant genera occur, *Crocodylus, Mecistops,* and *Osteolaemus* (subfamily Crocodylinae). Areas with higher PD and FD values comprise species with long thin/broad skulls, small/very large species when adults, fairly terrestrial species/highly aquatic^28,29^, living in sympatry in west-central Africa. Despite the same evolutionary lineages, morphological differences and habits make African species one of the higher FD values found in this study.

Crocodilians may play a key role as contributors to nutrient and energy cycling through cross-ecosystem movements worldwide, but there is a lack of research in this area^2^. Our exploratory findings show that the spatial distribution of crocodilians is positively correlated to nutrient retention, suggesting some competing hypotheses for the correlations found. These spatial patterns are better observed in Africa and the Americas, where higher values of FD are found in this study. Crocodilians of Asia and Oceania have a less spatial contribution (observed in our preliminary study) as ecological indicators of nutrient fluxes. However, they can play an important role in cross-ecosystem linkages through their food habits and dietary intakes, mainly in northeastern India, southeastern Asia, and northern Oceania which showed higher FD values. The interaction of crocodilians and nutrient retention is a preliminary investigation, and more complex causal mechanisms should also be considered in future studies.

Conservation practices ideally need evidence-based planning, but the fact that the species occurs in a PA, or whether their range has a good representation in the PA networks, does not imply the conservation of this individual species for example. Despite the distribution of half of the crocodilian species being well represented in the global PA networks, CR species do not have a good representation in the PA networks. Crocodilian management programs are also key in determining whether conservation efforts can be sustained^2^. Therefore, land use planning efforts prioritizing the establishment and maintenance of protected areas for crocodilian conservation, require private and governmental efforts to address the degradation of the natural environment and climate global changes.

Because of habitat loss, several crocodilian species are being hybridized^30^. The hybridization can result in decreased fitness of hybrids and distinctive genetic lineages^30^. Because of extensive hunting pressures from the middle of the nineteenth century, the population decline of crocodiles increased in Central and North America^31^. The crocodilian species like *Cr. rhombifer* and *Cr. moreletii* are suffering from these declines and hybridization processes in the Caribbean islands^11^. The species *Cr. acutus* is a marine species and co-occurs with these two species (i.e. *Cr. rhombifer* and *Cr. moreletii*), and if hybridized with these species^30,32^. Few populations of parental (non-admixed) *Cr. moreletii* remain in the wild^33^ and *Cr. rhombifer*, an endemic species in Cuba, is classified as CR by the IUCN Red List^4^, which is on the verge of extinction as a result of hybridization with *Cr. acutus*, both enhanced by human activities^32^. Our data show that the species *Cr. moreletii,* despite not being a threatened species, does not have a significant representation in the PA networks. Effective conservation and management strategies for crocodilians are crucial to maintaining their ecological and evolutionary values in land use planning^9,34^. We suggest that the establishment of new PA with a wide spatial range covering the species, and molecular efforts throughout its distribution are necessary for the conservation of this species.

In the Americas, three species do not have a good representation in the PA networks (*A. mississippiensis*, *Ca yacare,* and *Ca. latirostris*)*.* These species are classified as Least Concern (LC) by the IUCN Red List^4^. Empirical evidence on the *A. mississippiensis* in Florida demonstrated its ecosystem engineering role in creating microhabitats and foraging opportunities for plants and animals^35^. In the case of *Ca. latirostris* in South America, despite they have developed ability to colonize human-made habitats (e.g. small swamps in grasslands and secondary woodlands), is suffering continuous anthropogenic pressures in the Atlantic Forest^36,37^, one of the most threatened hotspots of the world^38,39^. Due to habitat loss, this species is increasingly isolated in small fragments, and many individuals that enter urban areas are exposed to various threats, such as hunting, climate change, invasive species, and pollution^37,40^. The species *Ca. yacare* occurs in the Pantanal biome in Brazil, which suffered one recent environmental disaster of anthropic origin (i.e. an intentional fire that reached huge proportions^41^). In addition to the death of animals in this biome, the catastrophic event triggered negative hydrological effects^42^, with many populations of *Ca. yacare* suffering from dehydration and infectious diseases in the worst affected areas, which raised the species’ mortality rate in this region^42^ (see Fig. S1). The loss of *Ca. yacare* in this region can disproportionately disrupt the ecosystem structure and function^43^, which may cause an ecological problem termed ‘trophic downgrading’ induced by lower-order consumers^44,45^. Although these species are not considered threatened, the low representation in the PA networks in areas so altered by human actions, makes them possibly threatened in the future due to climate changes associated with environmental degradation. Therefore, improving conservation actions for crocodilians in human-induced landscapes can avoid the extinction or decline in the population of crocodilian species with further impacts on food web dynamics and ecosystem stability.

In Asia and Oceania, the most alarming cases concern *G. gangeticus, Cr. palustris, A. sinensis,* and *Cr. halli* (see Fig. 3). The species *A. sinensis* is classified as CR by the IUCN Red List^4^. Because of decreasing of the population of *A. sinensis*, strategies for the conservation of this species are fundamental, thus some implications for the management of captive breeding have been implemented^46^. However, these individuals in captivity decrease the genetic diversity, resulting in a bottleneck effect^47^. It appears that isolated habitats may provide an environment with low exposure to pathogens, and genes have no motivation to change, causing an increase in similar genes across captive populations^47^. Our findings showed that *A. sinensis* has a representation non-existent in the PA networks, the establishment of protected areas in its range is essential for its conservation. In the case of the gharial (*G. gangeticus*—classified as CR by the IUCN Red List)^4^, the bottleneck effect occurs because habitat fragmentation isolated the populations, decreasing genetic diversity^48^. Previous conservation efforts in India concerning the threatened *G. gangeticus* benefit numerous other species at regional scales^49–51^. This suggests that for success in conserving crocodilians, the populations should be maintained in large PA networks, maximizing the representation of catchments and linear riparian systems, and maintaining their evolutionary history, genetic variability, and ecological functions.

Our results show that two threatened species that occur in India, have low representation in protected areas; CR species *G. gangeticus* with MPO = 0.515%, and VU species *Cr. palustris* with MPO = 0.685%. Unfortunately, we did not have access to PA networks in India which do not provide data to UNEP, WCMC & IUCN^52^. However, according to the Wild Life Institute of India (https://wii.gov.in/nwdc_aboutus), India has a PA network with 990 areas covering 5.27% of the country’s geographical area, with 106 National Parks and 565 Wildlife Sanctuaries, so the MPO results may be better for these species. Anyway, India enters Model 1(covering 20.45% of the priority area, see Table 2) and Model 3 (see Fig. 4) of conservation as one of the main areas for the establishment and maintenance of PA.

Our results show important patterns for the evaluation of species to be considered: (i) species threatened and not represented (*A. sinensis*, *Cr. palustris*, *G. gangeticus*) or with non-significant results (*Cr. mindorensis*, *Me. cataphractus*, *O. osborni*, *O. tetraspis*) in the current coverage of PA networks. These are the most alarming cases, the establishment or maintenance of PA is extremely important, and these species may be at serious risk of extinction; (ii) species not considered threatened by the IUCN, but threatened by anthropic actions in which our data show that they are not well represented or with non-significant results in the PA networks (*A. mississippiensis*, *Ca. yacare*, *Ca. latirostris*, *Cr. halli*, *Cr. morelleti*). These species can be considered as indicators for priority areas for the establishment of PA, to avoid a drastic decrease in their populations; (iii) threatened species with good representation in the PA networks (*Cr. acutus*, *Cr. suchus*, *Me. leptorhyncus*, *O. aftzelli*, *T. schlegelii*). The MPO analysis is based on the total area of occurrence of the species and their occurrence in the PA network, most of these species have a representativeness percentage with their coverage lower than 5% of MPO, recommending the establishment of new PA in their areas of occurrence connecting populations. In the case of *Cr. intermedius*, *Cr. rhombifer*, and *Cr. siamensis* have coverage above 5.5%, and almost all of their distribution is in PA, which denotes the importance of these PA and the connections between them are extremely important for the conservation of these CR species; and (iv) species not threatened and well represented in the network of protected areas (*Ca. crocodilus*, *Cr. johnsoni*, *Cr. niloticus*, *Cr. novaeguineae*, *Cr. porosus*, *Melanosuchus niger*, *P. palpebrosus*, *P. trigonatus*). These are the species of the least concern. However, it is important to clarify that our analysis for these species were based on the IUCN^4^ distribution data (i.e. polygons) and did not analyze their population size. Therefore, being well-represented does not exclude the possibility of being threatened by anthropic actions. In addition, they strongly contribute to the values of TD, FD and PD, and should be considered when choosing and maintaining priority areas.

We argue for the use of Model 1 as the main ecological indicator for crocodilian conservation on Earth (see details in Fig. 4, Table 2). However, Models 2 and 3 also show important areas for crocodilian conservation such as Central America, South America, west-central Africa, India, Sri Lanka, southeastern Asia, and northern Oceania (see Fig. 4). Model 2 and 3 also shows important areas for crocodilian conservation in Pantanal and Atlantic Forest biomes, in Brazil (Fig. 4), comprising two species of the genus *Caiman* (*Ca. yacare* and *Ca. latirostris*) that are not well represented in the PA networks. Another priority area for conservation in New Guinea and Papua New Guinea, where there the recently described species of the crocodile (*Cr. halli*) is not well represented in the PA networks. Global challenges for conserving the three dimensions of crocodilian diversity require more research and practical recommendations. However, the existing global PA networks have extreme importance for the conservation of the ecological and evolutionary values of crocodilians in the world.

It is important to emphasize that our model followed the distribution of species from the most recent literature or database found, and species such as *Me. catapractus* may be considered extinct or unconfirmed in some areas such as Benin and Nigeria. These unconfirmed areas can be critical areas for study focus and the establishment of protected areas for crocodilian species and should be considered. For species that occur in India such as *Cr. palustris* and China (*A. sinensis*), further confirmation of their distribution is needed as well as their presence in the PA networks.

Our models represent new conservation areas with a maximum relevance of evolutionary and ecological values for crocodilians and can help in the choice for the establishment or expansion of protected areas at different scales. We suggest the following steps in the application of the models: (i) choice of the model; (ii) presence of threatened species; (iii) confirmation of the presence of the species in the area; and (iv) ensuring that areas contain suitable environments for the species (i.e. maximizing the representation of catchments and linear riparian systems).

In the present study, we report key conservation areas that incorporate the three dimensions of crocodilian diversity (i.e. TD, FD and PD) under an integrative landscape plan. Our results emphasize global priority areas for crocodilian conservation, using evidence-based planning with multiple crocodilian diversity components. However, these findings demand political will and applied environmental actions in balance with social interests to reduce extinction risk and avoid species loss. In addition, maintenance and efforts in the PA networks may help prevent catastrophic encounters with crocodilians and humans. By using crocodilians as umbrella species for conservation, many species that co-occur with crocodilian species will benefit. Therefore, using multiple biodiversity components in balance with the landscape and their potential threats is essential to improve future strategies in designing effective conservation models.

## Methods

### Spatial data

We created an updated database with geographic distribution maps of the IUCN Red List, version 2022-1^4^ for all species of crocodilians distributed on a global scale, and Smolenski et al.^53^, Shirley et al.^54,55^, Murray et al.^56^, Hekkala et al.^57^, Mobaraki et al.^58^, Cunninghan et al.^59^, and Platt et al.^60^ for actualized distributions of *Osteolaemus*, *Mecistops*, *Crocodylus halli, Cr. novaguinae*, *Cr. palustris, Cr. niloticus*, *Cr. suchus,* and *Cr siamensis* respectively. We use the most up-to-date distribution of the species. Then, we created a presence/absence matrix, superimposing the species distribution data on a grid system with a spatial resolution of 0.5 degrees, using ArcGIS Pro software^61^. In total, we assessed the geographical ranges of 28 crocodilian species covered by our grid system of 38,974 grid cells.

### Calculating taxonomic, functional and phylogenetic diversity

We calculated and mapped taxonomic diversity (TD) by summing the number of crocodile species in each cell of the world grid. We calculated the FD of Crocodilians through a database of Griffith et al.^28^ and the present study totalized 13 functional traits divided into five categories of morphology, life history, and behavior characteristics (e.g.^25,28,62^). The functional traits were categorized as (1) body size (largest male size and female size at maturity); (2) habitat type (generality, salt tolerance, and terrestriality); (3) tolerance to extreme climates (aestivation and brumation); (4) potential to act as ecosystem engineers (ability to dig burrows); (5) activity (day, night, and both); (6) diet/foraging strategy (diet generality, skull shape, bite force); (7) Reproduction (nest type, relative clutch mass). The skull shape was combined as a single trait (see Griffith et al.^28^). For further details of specific functions and ecosystem-supporting services of each one of the functional traits assessed, see Griffith et al.^28^ and Supplementary Tables S1 and S2.

We followed the protocol proposed by Petchey and Gaston^16^ to calculate FD: (1) construction of a species-trait matrix; (2) conversion of the species-trait matrix into a distance matrix; (3) clustering distance matrix into a dendrogram (UPGMA); and (4) calculating functional diversity by summing dendrogram branch lengths of species community. To create distance matrices, we used the method Gower distance^63^.

We based the phylogenetic distance on the phylogeny proposed by Colston et al.^64^ which contains 27 of the species. We used the software R^65^ for the reconstruction of the phylogenetic tree using the package ‘ape’. For phylogenetic analysis, we used Faith’s PD index^66^ because has appropriate ways of accounting for relatedness between taxa and evolutionary history in a conservation context^67^. Faith’s PD index comprises the sum of the branch lengths of the phylogenetic tree of all species assessed and is often used in the assessment of phylogenetic diversity of co-occurring species (e.g.^68–70^). The analyses were done for each grid cell of 0.5 degrees (38,974 grid cells). We verified whether FD and PD were influenced by species richness^71^, using independent swap null models^72^.

The values provided by such models are more sensitive to preserving both site diversity and species frequency of occurrence while randomizing the pairs of species/sites, which ensures that patterns of trait assembly do not simply reflect the differential occurrence of species^1,72^. The null model is independent of the species richness of an assemblage^71^, which provides expected values at different species richness levels^73^. Hence, we tested if the functional and phylogenetic diversity were higher, equal, or lower than expected by chance for each grid cell (random or non-random pattern), assuming a random distribution in which every species could occupy any grid cell in the biome. We computed 1000 replicates of random remaining PD and FD, allowing us to obtain a *p*-value of predicted PD and FD as compared to the distribution of the random replicates. We correlated the values obtained for TD, FD and PD in each grid cell using simple linear correlation models (normality was evaluated using the Shapiro–Wilk test). All analyses were performed using the packages ‘ade4’, ‘picante’, ‘FD’, and ‘vegan’ through the R software^65^. The Phylogenetic Tree and Functional Tree are available in Supplementary Fig. S2.

### Estimating crocodilians vs nutrient retention relationships

To estimate the potential correlations of crocodilians to ecosystem services of nutrient retention, we used the global data provided by Chaplin-Kramer et al.^74^ and map it on our grid system under a spatial resolution of 0.5°. The nutrient retention data were provided from the InVEST (Integrated Valuation of Ecosystem Services and Tradeoffs) nutrient delivery model^75^, which is used to map nutrient sources from watersheds and their transport to the streams based on land cover. Nutrient retention maps reflect the pollution avoided in water-related ecosystems and are assessed by subtracting nitrogen load and nitrogen export values for water purification services. For these analyses, we used correlation linear models between TD, FD and PD vs. nutrient retention for Americas, Africa/Middle East, and Asia/Oceania, through the package ‘vegan’, in the R software^65^.

### Calculating the effectiveness of the existing PA networks

To compile a list of species supported by the PA networks available from UNEP-WCMC & IUCN^52^, we compiled spatial data on the distribution of PA networks in the world within the IUCN Red List categories (i.e. I to IV)^4^, which represent the National, State, and Municipal reserves, totaling 38,010 PA. We then superimposed the species distribution data on a gridded representation of the PA separately according to region, Americas (included South, Central and North America, 8223 PA), Africa (included all Africa and the Middle East, 1217 PA), and Asia/Oceania (included all Asia and Oceania, 28,579 PA) for each grid cell of resolution of 0.5 degrees. In preparation for the subsequent analyses, we used ArcGIS Pro software^60^ to create a presence/absence matrix of species per grid cell Americas (11,358 grid cells), Africa/Middle East (12,753 grid cells), and Asia/Oceania (14,863 grid cells) a matrix describing the percentage of the grid cell occupied by PA.

To demonstrate the level of representativeness of crocodilian species in the existing PA networks in the world, we calculated the Mean Percentage Overlap—MPO^76,77^. The MPO corresponds to the mean percentage of spatial overlap between the units in which the species occurs in the studied area and the protected areas. We obtained the spatial overlap (%) of each cell of the study area with the polygons of the PA networks. Then, we used null models to test if the level of the MPO of each species was significantly different (lower or higher) than expected by chance, considering the number of occupied cells of each species (i.e. range size). For that, we used the software R^64^ to compare the observed MPO value of each species with MPO values obtained from 1000 randomizations using a significance level of *p* < 0.05.

### Mapping the priority conservation areas

To map the priority conservation areas, we implemented three dimensions of the crocodilian diversity (TD, PD and FD), and the presence of threatened species following the IUCN Red List criteria^4^. For the conservation status of threatened species, we used the three major categories of the Red List assessments (CR = Critically Endangered, EN = Endangered, and VU = Vulnerable)^4^. Then, we run three prioritization models based on different levels of complementary scenarios adapted from Campos et al.^18^, as follows:

$${\text{Model1}}_{{({\text{90}}\% )}} = \left\{ {{\text{FD}} \ge \left[ {(0.{\text{9}}((\sum\nolimits_{{{\text{i}} = 0}}^{{\text{n}}} {{\text{FD}}} ){\text{/N}})){\text{/0}}.{\text{5}}} \right] + {\text{PD}} \ge \left[ {\left( {0.{\text{9}}\left( {\left( {\sum\nolimits_{{{\text{i}} = 0}}^{{\text{n}}} {{\text{PD}}} } \right){\text{/N}}} \right)} \right){\text{/0}}.{\text{5}}} \right]{\text{ + TD}} \ge \left[ {\left( {0.{\text{9}}((\sum\nolimits_{{{\text{i}} = 0}}^{{\text{n}}} {{\text{TD}}} ){\text{/N}})} \right){\text{/0}}.5} \right] + {\text{CR}}} \right\},$$

$${\text{Model2}}_{{({\text{70}}\% )}} = \left\{ {{\text{FD}} \ge \left[ {\left( {0.{\text{7 }}\left( {\left( {\sum\nolimits_{{{\text{i}} = 0}}^{{\text{n}}} {{\text{FD}}} } \right){\text{/N}}} \right)} \right){\text{/0}}.{\text{7}}} \right] + {\text{PD}} \ge \left[ {\left( {0.{\text{7 }}\left( {\left( {\sum\nolimits_{{{\text{i}} = 0}}^{{\text{n}}} {{\text{PD}}} } \right){\text{/N}}} \right)} \right){\text{/0}}.{\text{5}}} \right] + {\text{TD}} \ge {\text{ }}\left[ {\left( {0.{\text{7 }}\left( {\left( {\sum\nolimits_{{{\text{i}} = 0}}^{{\text{n}}} {{\text{TD}}} } \right){\text{/N}}} \right)} \right){\text{/0}}.5} \right] + {\text{EN}}} \right\} - {\text{Model1}}_{{({\text{90}}\% )}} ,$$

$${\text{Model3}}_{{({\text{50}}\% )}} {\text{ = }}\left\{ {{\text{FD}} \ge \left[ {\left( {0.{\text{5}}\left( {\left( {\sum\nolimits_{{{\text{i}} = 0}}^{{\text{n}}} {{\text{FD}}} } \right){\text{/N}}} \right)} \right)} \right]{\text{ + PD}} \ge \left[ {\left( {0.{\text{5}}\left( {\left( {\sum\nolimits_{{{\text{i}} = 0}}^{{\text{n}}} {{\text{PD}}} } \right){\text{/N}}} \right)} \right)} \right]{\text{ + TD}} \ge {\text{ }}\left[ {\left( {0.{\text{5 }}\left( {\left( {{\text{ }}\sum\nolimits_{{{\text{i}} = 0}}^{{\text{n}}} {{\text{TD}}} } \right){\text{/N}}} \right)} \right)} \right] + {\text{VU}}} \right\} - {\text{Model2}}_{{({\text{70}}\% )}} .$$

Model 1 identifies areas that hold very high priority for conservation because of levels of per-cell FD, PD, and TD ≥ 90% (0.9) of the total observed (N), and the presence of CR species^4^; Model 2 identifies areas that hold high levels of per-cell FD, PD, and TD ≥ 70% (0.7) of the total observed (N), and the presence of EN species^4^; Model 3 identifies areas that hold medium levels of per-cell FD, PD, and TD ≥ 50% (0.5) of the total observed (N), and the presence of VU species^4^.

The main reason for this modeling approach was to select areas from medium to very high priority, leaving out low-priority regions. In this context, these models allow practical recommendations for crocodilian conservation efforts and provide a spatial prioritization ranking worldwide.

## Supplementary Information

Supplementary Information.

## Data Availability

All data generated or analyzed during this study are included in this article and its Supplementary Information files. No live animals were used in this study.
