# Supplementary material for: Global conservation prioritization areas in three dimensions of crocodilian diversity
Source: Sci Rep. 2023 Feb 13;13:2568. doi: 10.1038/s41598-023-28413-6 (PMC9925794; doi:10.1038/s41598-023-28413-6)
Supplement: Supplementary file 1 — Supplementary Information. [file 41598_2023_28413_MOESM1_ESM.doc]

**Supplementary information**

**Global conservation prioritization areas in three dimensions of crocodilian diversity**

**Fig. S1.** *Caiman yacare* died due to dehydration in South America.

**Fig. S2.** Phylogenetic and functional tree.

**Table S1.** List of studied crocodilian species and their respective functional traits.

**Table S2.** Specific functions, the ecosystem supporting services, and references related to the crocodilian's functional traits are assessed in the World.


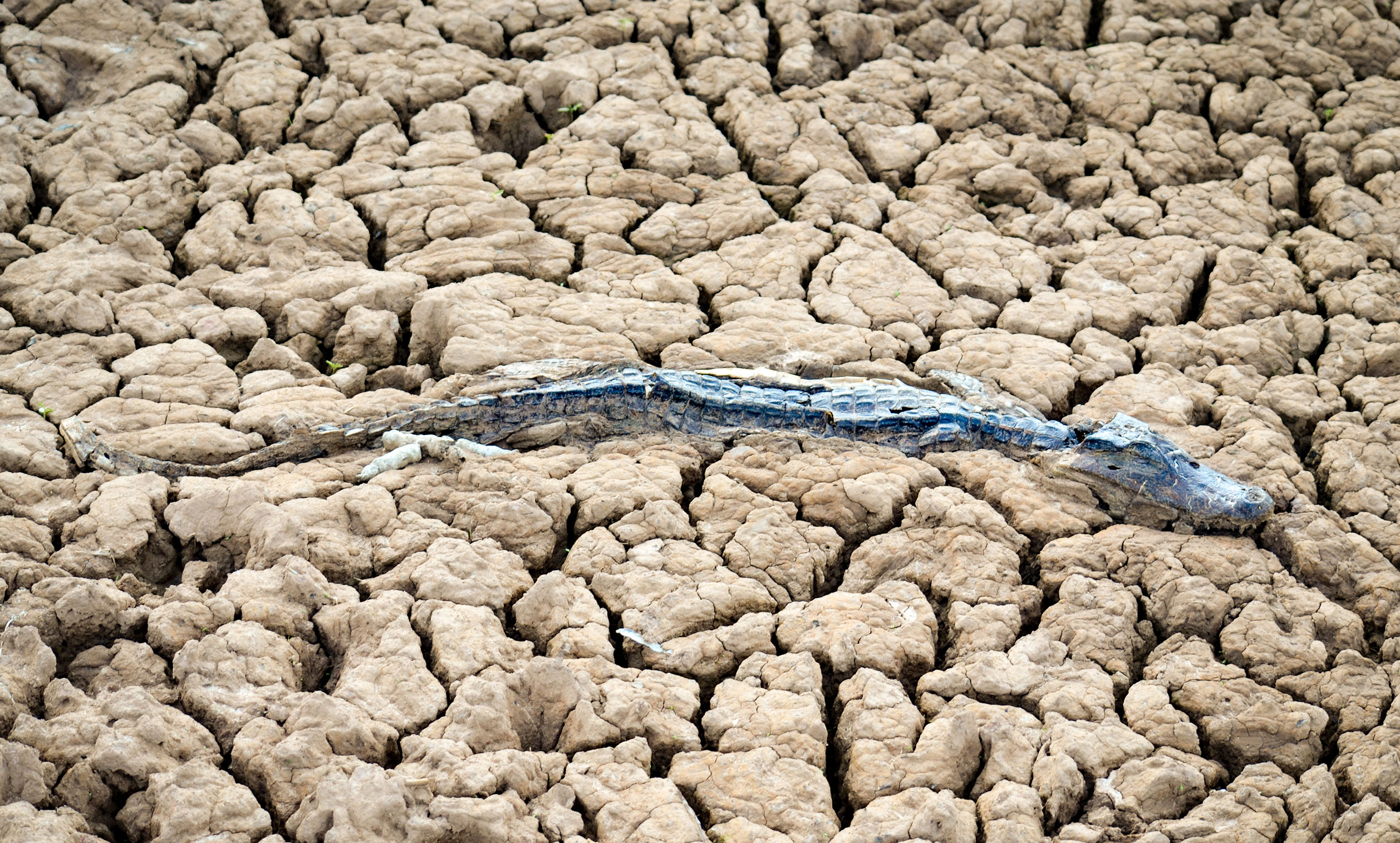


Fig. S1. *Caiman yacare* dead due to dehydration in Brazilian Pantanal in South America, Brazil. Photo by T. Silva-Soares.


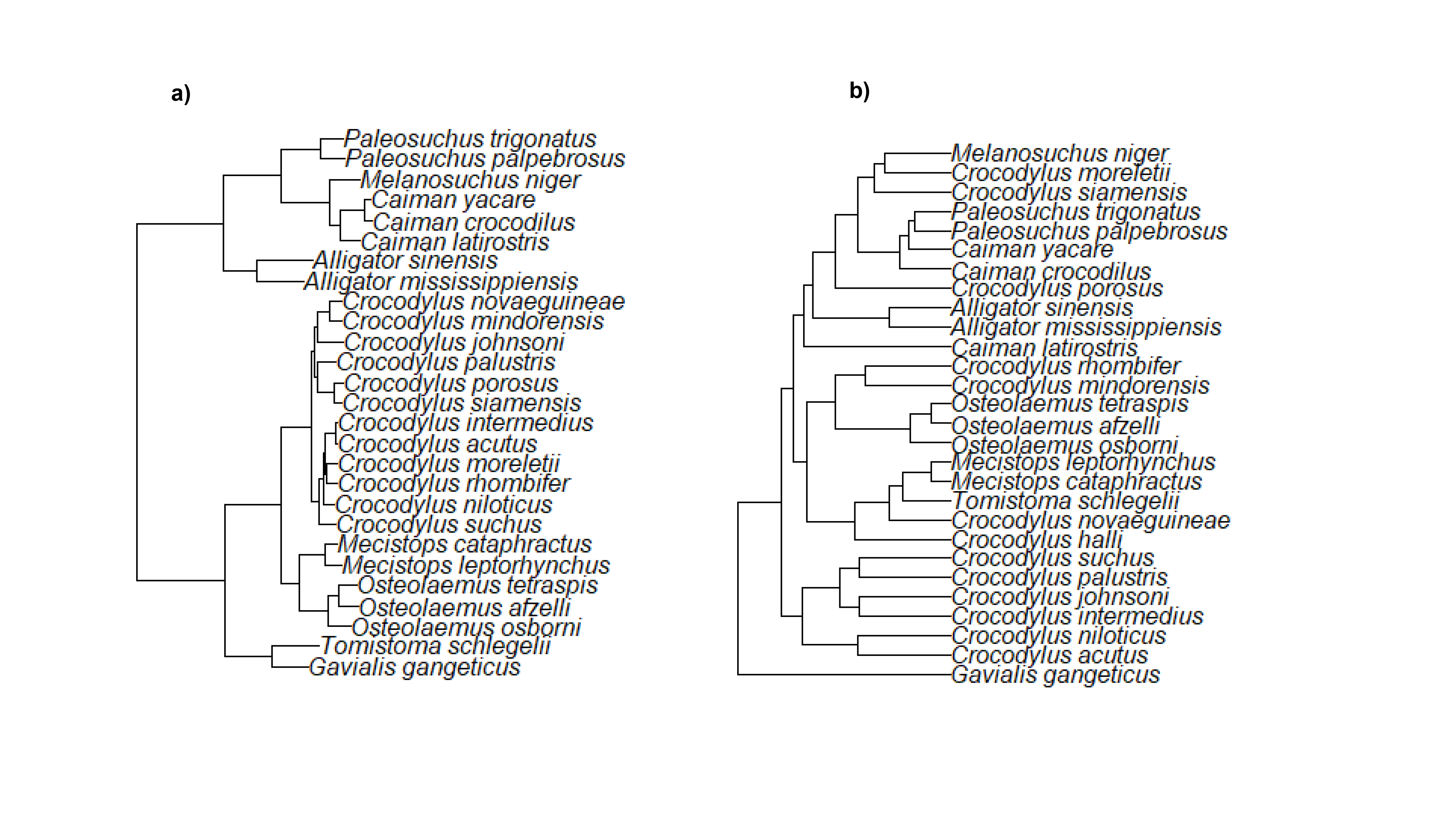


Fig S2. Phylogenetic tree a) and Functional tree b) used for analyses.

**Table S1.**  List of studied crocodilian species and their respective functional traits. For more details about functional traits body size, habitat type, tolerance to extreme climates, potential to act as ecosystem engineers, foraging strategy, and reproduction see Griftti et al^19^.

| **Species** | **Activity** | **Diet generalism** | **References** |
| --- | --- | --- | --- |
| *Alligator mississippiensis* (Daudin, 1801) | Night and day | Fishes, arthropods, mammals, amphibians, reptiles, birds | ^1^; ^2^; ^3^; ^4^; ^5^; ^6^ |
| *Alligator sinensis* Fauvel, 1879 | Night and day | Fishes, molluscs, mammals, birds | ^2^; ^3^; ^4^; ^5^; ^6^; ^7^ |
| *Caiman crocodilus* (Linnaeus, 1758) | Night and day | Fishes, molluscs, arthropods, mammals, amphibians, reptiles, birds | ^2^; ^3^; ^4^; ^5^; ^6^; ^8^; ^9^ |
| *Caiman latirostris* (Daudin, 1801) | Night and day | Fishes, molluscs, arthropods, mammals, amphibians, reptiles, birds | ^2^; ^3^; ^4^; ^5^; ^6^; ^10^ |
| *Caiman yacare*  (Daudin, 1801) | Night | Fishes, molluscs, arthropods, mammals, reptiles, birds | ^2^; ^3^; ^4^; ^5^; ^11^ |
| *Crocodylus acutus* (Cuvier, 1807) | Night | Fishes, arthropods, reptiles, birds | ^2^; ^3^; ^4^; ^5^ |
| *Crocodylus halli* Murray, Russo, Zorilla & Mcmahan, 2019 | Night | Fishes, arthropods, amphibian, reptiles, birds | ^2^; ^3^; ^4^; ^12^ |
| *Crocodylus intermedius* (Graves, 1819) | Night | Fishes, arthropods, mammals, reptiles, birds | ^2^; ^3^; ^4^; ^5^ |
| *Crocodylus johnsoni*  Krefft, 1873 | Night | Fishes, arthropods, moluscs, mammals, amphibians, reptiles, birds | ^2^; ^3^; ^4^; ^5^ |
| *Crocodylus mindorensis* Schmidt, 1935 | Night | Fishes, arthropods, moluscs, mammals, reptiles, birds | ^2^; ^3^; ^4^; ^5^; ^6^ |
| *Crocodylus moreletii* (Duméril & Bibron, 1851) | Night | Fishes, moluscs, mammals, reptiles, birds | ^2^; ^3^; ^4^; ^5^ |
| *Crocodylus niloticus* Laurenti, 1768 | Night | Fishes, arthropods, molluscs, mammals, amphibians, reptiles, birds | ^2^; ^3^; ^4^; ^5^ |
| *Crocodylus novaeguineae*  Schmidt, 1928 | Night | Fishes, arthropods, amphibians, reptiles, birds | ^2^; ^3^; ^4^; ^5^ |
| *Crocodylus palustris* (Lesson, 1831) | Night | Fishes, arthropods, molluscs, mammals, amphibians, reptiles, birds | ^2^; ^3^; ^4^; ^5^ |
| *Crocodylus porosus* Schneider, 1801 | Night | Fishes, arthropods, mammals, amphibians, reptiles, birds | ^2^; ^3^; ^4^; ^5^ |
| *Crocodylus rhombifer* (Cuvier, 1807) | Night and day | Fishes, mammals, reptiles | ^2^; ^3^; ^4^; ^5^; ^6^ |
| *Crocodylus siamensis* Schneider, 1801 | Night | Fishes, mammals, amphibians, reptiles, birds | ^2^; ^3^; ^4^; ^5^ |
| *Crocodylus suchus* Geoffroy Saint-Hilaire, 1807 | Night and day | Fishes, arthropods, molluscs, mammals, amphibians, reptiles, birds | ^2^; ^3^; ^4^; ^5^ |
| *Gavialis gangeticus* (Gmelin, 1789) | Night | Fishes, arthropods, mammals, amphibians | ^2^; ^3^; ^4^; ^5^ |
| *Mecistops cataphractus* Cuvier, 1825 | Night | Fishes, mammals, reptiles, birds | ^2^; ^4^; ^5^; ^13^ |
| *Mecistops leptorhynchus* (Bennett, 1835) | Night | Fishes, mammals, reptiles, birds | ^2^; ^3^; ^4^; ^13^ |
| *Melanosuchus niger* (Spix, 1825) | Night | Fishes, arthropods, molluscs, mammals, reptiles | ^2^; ^3^; ^4^; ^5^; ^6^; ^8^; ^14^ |
| *Osteolaemus aftezelli* Lilljeborg, 1867 | Night | Fishes, arthropods, amphibians | ^2^; ^3^; ^4^; ^5^ |
| *Osteolaemus osborni* Schmidt, 1919 | Night | Fishes, arthropods, amphibians | ^2^; ^3^; ^4^; ^5^ |
| *Osteolaemus tetraspis* Cope, 1861 | Night | Fishes, arthropods, amphibians | ^2^; ^3^; ^4^; ^5^ |
| *Paleosuchus palpebrosus*  (Cuvier, 1807) | Night | Fishes, arthropods, molluscs, mammals, amphibians, reptiles, birds | ^2^; ^3^; ^4^; ^5^; ^6^; ^15^ |
| *Paleosuchus trigonatus* (Schneider, 1801) | Night | Fishes, arthropods, molluscs, mammals, amphibians, reptiles, birds; aquatic plants | ^2^; ^3^; ^4^; ^5^; ^6^; ^8^; ^16^ |
| *Tomistoma schlegelii* (Müller, 1838) | Night | Fishes, arthropods, mammals, reptiles, birds | ^2^; ^3^; ^4^; ^5^ |

**Table S2.** Specific functions, the ecosystem supporting services, and references related to the crocodilian functional traits assessed in the World.

For more details about functional traits body size, habitat type, tolerance to extreme climates, potential to act as ecosystem engineers, foraging strategy, and reproduction see Griftti et al^19^.

| **Functional traits** | **Specific**  **functions** | **Ecosystem**  **supporting services** | **References** |
| --- | --- | --- | --- |
| Activity  (nocturnal, diurnal, and both) | Dispersal ability, predator-prey relationships, sexual selection | Trophic transfer, nutrient cycling | ^2^; ^3^; ^17^; ^18^ |
| Diet  (fishes, annelids, arthropods, molluscs, mammals, amphibians, birds, and aquatic plant) | Predator-prey relationships, dispersion of seeks | Trophic transfer, nutrient cycling | ^2^; ^3^; ^17^; ^18^ |

##

**References**

19 Griffith, P., Lang, J. W., Turvey, S. T. & Gumbs, R. Data from: Using functional traits to identify conservation priorities for the world's crocodylians. *Zenodo*. https://doi.org/10.5281/zenodo.6645415 (2022).
